# Generalized Solutions of Parrondo's Games

**DOI:** 10.1002/advs.202001126

**Published:** 2020-11-07

**Authors:** Jin Ming Koh, Kang Hao Cheong

**Affiliations:** ^1^ Science, Mathematics and Technology Cluster Singapore University of Technology and Design (SUTD) 8 Somapah Rd Singapore S487372 Singapore; ^2^ California Institute of Technology Pasadena CA 91125 USA; ^3^ SUTD‐Massachusetts Institute of Technology International Design Centre 8 Somapah Rd Singapore S487372 Singapore

**Keywords:** analytical methods, Brownian ratchets, game theory, generalized solutions, noise, non‐linear dynamics, Parrondo's paradox

## Abstract

In game theory, Parrondo's paradox describes the possibility of achieving winning outcomes by alternating between losing strategies. The framework had been conceptualized from a physical phenomenon termed flashing Brownian ratchets, but has since been useful in understanding a broad range of phenomena in the physical and life sciences, including the behavior of ecological systems and evolutionary trends. A minimal representation of the paradox is that of a pair of games played in random order; unfortunately, closed‐form solutions general in all parameters remain elusive. Here, we present explicit solutions for capital statistics and outcome conditions for a generalized game pair. The methodology is general and can be applied to the development of analytical methods across ratchet‐type models, and of Parrondo's paradox in general, which have wide‐ranging applications across physical and biological systems.

## Graphical abstract

The game‐theoretic Parrondo's paradox refers to the attainment of winning outcomes by alternating between losing strategies. Closed‐form solutions are presented for capital statistics in a stochastically mixed game pair with drawing outcomes, and in a further generalization with independently tunable game branches; the solutions accommodate arbitrary symmetry modulus *M*. The approach can be adapted to other ratchet‐type models.

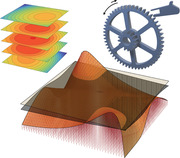

## 1 Introduction

Particles can exhibit bulk drift when exposed to asymmetric periodic potentials, even when the space‐averaged force is zero or in an opposing direction^[^ ^1^, ^2^, ^3^ ^]^; underlying the unexpected motion is an agitation‐ratcheting mechanism, formed from alternating diffusive and localizing temporal phases as the potential oscillates. This has accordingly been termed the flashing Brownian ratchet, in similarity to the Brownian ratchet‐and‐pawl system of Smoluchowski and Feynman,^[^ ^4^, ^5^ ^]^ and has been applied successfully in the control of particle ensembles.^[^ ^6^ ^]^ An abstraction of the phenomenon is Parrondo's paradox, in which switching between two game‐theoretic losing strategies in a random or deterministic order can result in winning outcomes.^[^ ^7^ ^]^

Molecular motors and enzyme transport had been analyzed through Brownian ratchet models^[^ ^8^, ^9^, ^10^ ^]^; more recently, the broader class of Parrondo's paradox has been used to describe a large range of phenomena, including stability in mixed chaotic systems,^[^ ^11^, ^12^ ^]^ unexpected drifts in granular flow^[^ ^13^ ^]^ and switched diffusion processes,^[^ ^14^ ^]^ and entropic behaviour in information thermodynamics.^[^ ^15^, ^16^ ^]^ Quantum paradoxical systems have also been studied, some suggesting implications on quantum information processing,^[^ ^17^ ^]^ and algorithms exploiting the paradox have been devised for engineering optimization.^[^ ^18^, ^19^ ^]^ In biophysics, evolutionary processes,^[^ ^20^, ^21^, ^22^, ^23^, ^24^, ^25^ ^]^ population biology,^[^ ^26^ ^]^ ecological dynamics,^[^ ^27^, ^28^, ^29^ ^]^ cellular machinery,^[^ ^30^, ^31^, ^32^ ^]^ and social behavior^[^ ^33^, ^34^, ^35^ ^]^ have all been linked to the paradox, and a degree of universality across scales is suspected.^[^ ^36^ ^]^ The seminal results in genetics include the possibility of proliferation and fixation of lower‐fitness autosomal alleles amidst antagonistic selection and epistasis,^[^ ^37^ ^]^ and selection for random phase variation^[^ ^23^, ^38^ ^]^ and phenotypic switching^[^ ^24^, ^25^ ^]^ in microorganisms, despite the strategy being likely locally maladaptive; in ecological systems, environmental fluctuations have been shown to enable the persistence of rare species in invaded habitats.^[^ ^39^ ^]^ The framework of Parrondo's games has even seen applications in the modeling and control of cancer tumours, to varying degrees of approximation.^[^ ^40^, ^41^, ^42^ ^]^

Minimally, the classical Parrondo's paradox can be represented by a pair of games^[^ ^43^ ^]^—the first a coin‐flipping game, analogous to a linear potential supporting diffusive spread in the flashing Brownian ratchet, and the second having two branches, one played when the capital is a multiple of a modulus M and the other played otherwise, analogous to an asymmetric periodic potential supporting localization of particle density. Stochastically mixing the games can produce winning outcomes (positive capital drift) despite both being losing individually. Despite the significant research interest, current descriptions of the properties of the paradox is incomplete.^[^ ^17^, ^36^, ^43^, ^44^ ^]^ Perturbative and asymptotic analyses have been presented,^[^ ^45^, ^46^ ^]^ alongside simulational studies,^[^ ^47^ ^]^ but explicit solutions for capital statistics for arbitrary modulus M have not been reported; known solutions for natural generalizations of the games also remain sparse.

In this paper, we present explicit closed‐form solutions for capital mean and variance in Parrondo's games generalized to include drawing outcomes; solutions for a further generalization into M‐branch games are also derived, for arbitrary probability configuration and M. The boundaries of winning, drawing, and losing parameter spaces are similarly found in closed‐form. These results are exact in steady‐state, and accurate to O(1/n) for n rounds in transient conditions; extensive verification against simulations is provided. This work essentially gives a complete solution to single‐agent capital‐dependent Parrondo's games, extensible to the multitude of variants modelable on discrete‐time Markov chains.

## 2 Generalized Game Pair

A generalized capital‐dependent Parrondo's game structure with drawing outcomes is first considered. In game A, winning, drawing, and losing outcomes, respectively, occur with probability p, r, and q=1−p−r in each round; in game B, they respectively occur with probability $p_{1}$, $r_{1}$, and $q_{1}=1-p_{1}-r_{1}$ when capital c is divisible by M≥3 and $p_{2}$, $r_{2}$, and $q_{2}=1-p_{2}-r_{2}$ otherwise. Stochastic mixing is controlled by parameter γ, reflecting probabilities γ and 1−γ of playing game A and B, respectively, at each round. Each winning and losing outcome results in a unit capital increment ($\eta_{p}=1$) and decrement ($\eta_{q}=-1$), respectively, and each draw results in unchanged capital ($\eta_{r}=0$). Lose–win ratios are ϕ=q/p, $\phi_{1}=q_{1}/p_{1}$, and $\phi_{2}=q_{2}/p_{2}$.

### 2.1 Conditions

The conditions for winning, fair, and losing outcomes can be deduced by considering the probability of reaching zero capital. With mixed transition probabilities $s_{i}^{\prime }=\gamma s+\left(1-\gamma \right)s_{i}$ and mixed lose–win ratios $\phi_{i}^{\prime }=q_{i}^{\prime }/p_{i}^{\prime }$ for s∈{p,r,q} and i∈{1,2}, the conditions are remarkably simple

(1) $$\begin{array}{cccc} & \text{winning} & & \text{if}\;\varphi <1\\ & \text{fair} & & \text{if}\;\varphi =1\\ & \mathrm{losing} & & \text{if}\;\varphi >1 \end{array}$$

where $\varphi =\varphi_{R}=\phi_{1}^{\prime }\phi_{2}^{\prime M-1}$ for stochastically mixed games. For pure game A, γ=1, reducing $s_{i}^{\prime }=s$, and $\varphi_{A}=\phi$; whereas for pure game B, γ=0, reducing $s_{i}^{\prime }=s_{i}$, and $\varphi_{B}=\phi_{1}\phi_{2}^{M-1}$. A derivation of this result is given in Appendix A1.

### 2.2 Capital Statistics

We now use an explicit Markov chain formulation, seeking analytical solutions for expected capital μ(n) and capital variance $\sigma^{2}\left(n\right)$ at round n. Capital states $\left\{S_{1},S_{2},\ldots ,S_{M}\right\}$ satisfying c≡(i−1)modM for state $S_{i}$ are considered. The distribution between these states $\omega =[\omega_{1}\;\omega_{2}\;\ldots \;\omega_{M}]$ at a game round can be found from that of the previous $\omega_{-1}$ as $\omega =\omega_{-1}H$ where H is the transition matrix, defined by

(2) $$H_{\mathrm{ij}}=\left\{\begin{array}{cc} p_{2-\delta_{1i}}^{\prime } & \text{if}\;j\equiv (i\;\mathrm{mod}\;M)+1\\ q_{2-\delta_{1i}}^{\prime } & \text{if}\;i\equiv (j\;\mathrm{mod}\;M)+1\\ r_{2-\delta_{1i}}^{\prime } & \text{if}\;i=j\\ 0 & \text{otherwise} \end{array}\right.$$

where $\delta_{ij}$ is the Kronecker delta. The corresponding payoff matrix W is of similar form as H but with $s_{i}\to \eta_{s}$ for s∈{p,r,q} and i∈{1,2}, and Q=H∘W is defined, where ∘ denotes the Hadamard product. The stationary distribution is characterized by the eigenvector equation ω=ωH, which can be converted to a coupled recurrence system and solved with a normalization constraint (see Appendix A2) to yield the solution

(3) $$\begin{array}{ccc} \omega_{m} & = & \;{\left(p_{2}^{\prime }/p_{1}^{\prime }\right)}^{\delta_{1m}}\left(\alpha \phi_{2}^{\prime -m}+\beta \right)\\ \alpha & = & \;\left(p_{2}^{\prime }-q_{2}^{\prime }\right)\left(p_{2}^{\prime }q_{1}^{\prime }-p_{1}^{\prime }q_{2}^{\prime }\right)\phi_{2}^{\prime }/\lambda \\ \beta & = & \;\left(p_{2}^{\prime }-q_{2}^{\prime }\right)\left(p_{1}^{\prime }\phi_{2}^{\prime -M}q_{2}^{\prime }-p_{2}^{\prime }q_{1}^{\prime }\right)/\lambda \\ \lambda & = & \;p_{2}^{\prime }q_{2}^{\prime }\left(\phi_{2}^{\prime -M}-1\right)\left[\left(p_{2}^{\prime }-p_{1}^{\prime }\right)-\left(q_{2}^{\prime }-q_{1}^{\prime }\right)\right]\\ & & -\;M\left(p_{2}^{\prime }-q_{2}^{\prime }\right)\left(p_{2}^{\prime }q_{1}^{\prime }-p_{1}^{\prime }q_{2}^{\prime }\phi_{2}^{\prime -M}\right) \end{array}$$

for m∈[1,M]. The average outcome probabilities $\bar{p}$, $\bar{r}$, and $\bar{q}$, are

(4) $$\bar{s}=\omega_{1}s_{1}^{\prime }+\left(1-\omega_{1}\right)s_{2}^{\prime }$$

for s∈{p,r,q}. The steady‐state per‐round change in expected capital Δμ and the capital mean μ(n) are therefore

(5a) $$\begin{array}{cc} \Delta \mu & =E\left[\eta \right]=\bar{p}-\bar{q} \end{array}$$

(5b) $$\begin{array}{cc} \mu (n) & =n\Delta \mu \end{array}$$

Equivalently, Δμ=ωQℓ where ℓ is a column vector of ones; an alternative derivation via the capital distribution is given in Appendix A3. Next, obtaining the capital variance $\sigma^{2}\left(n\right)$ necessitates computing the fundamental matrix of the Markov chain,^[^ ^45^, ^48^ ^]^ $Z=I+\sum_{n=1}^{\infty }\left(H^{n}-\Omega \right)={\left(I-H+\Omega \right)}^{-1}$, where Ω is the limiting matrix comprising ω as rows and identity Z(I−H)=I−Ω applies. A similar solving method can be used; the derivation and full solution for Z is detailed in Appendix A4. The steady‐state per‐round change in capital variance $\Delta \sigma^{2}$ and capital variance $\sigma^{2}\left(n\right)$ are

(6a) $$\begin{array}{cc} \Delta \sigma^{2} & =E\left[\eta^{2}\right]-E{\left[\eta \right]}^{2}+2\sum_{m=1}^{\infty }\mathrm{cov}\left(\eta ,\eta_{m}\right)\\ & =\bar{p}+\bar{q}-{\left(\bar{p}-\bar{q}\right)}^{2}+2d \end{array}$$

(6b) $$\begin{array}{cc} \sigma^{2}\left(n\right) & =n\Delta \sigma^{2} \end{array}$$

where $\mathrm{cov}(\eta ,\eta_{m})$ is the covariance in capital change between m game rounds and d=ωQ(Z−Ω)Qℓ. This yields

(7) $$\begin{array}{cc} d= & \;\left[\left(p_{1}^{\prime }-q_{1}^{\prime }\right)-\left(p_{2}^{\prime }-q_{2}^{\prime }\right)\right]\sum_{i=1}^{M}\left(p_{2-\delta_{i2}}^{\prime }\omega_{i_{-}}-q_{2-\delta_{iM}}^{\prime }\omega_{i_{+}}\right)\left(Z_{i1}-\omega_{1}\right) \end{array}$$

where we denote $i_{\pm }=1+\left(i-1\pm 1\right)\;\mathrm{mod}\;M$ as the modular successor and predecessor of index i, and $\omega_{m}$ is given in Equation (3). Substituting d into Equation (6) completes the closed‐form solution for $\sigma^{2}\left(n\right)$. In summary, we have analytical solutions for parameter space boundaries in Equation (1), and capital statistics μ(n) and $\sigma^{2}\left(n\right)$ in Equations (5) and (6). We note μ(n) and $\sigma^{2}\left(n\right)$ are constructed from exact steady‐state Δμ and $\Delta \sigma^{2}$, and therefore neglect initial transients in the game; accuracy is O(1/n) when transients are present.

### 2.3 Validation

**Figure 1:**
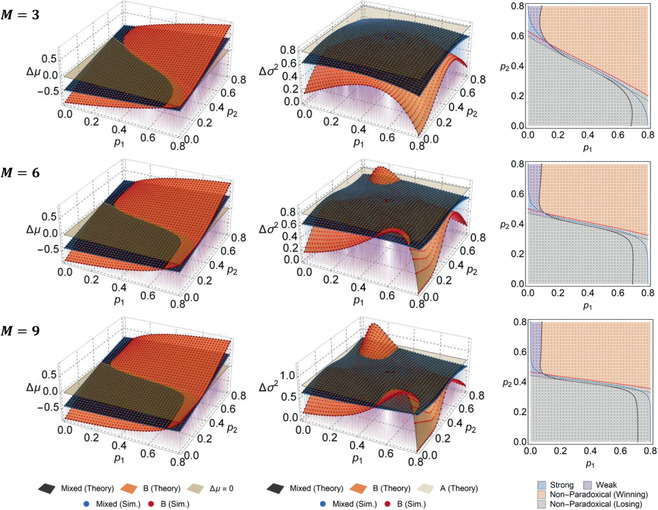
Comparison of Δμ and $\Delta \sigma^{2}$ from theory and simulations across $p_{1}-p_{2}$ parameter space, for differing M. Relative error between theory and simulation is <0.1% throughout. Phase diagrams are also shown; background colors represent theory and dots represent simulation. Dotted lines denote theoretical boundaries, for $\Delta \mu_{R}=\Delta \mu_{B}$ (black), $\Delta \mu_{R}=\Delta \mu_{A}$ (gray), $\Delta \mu_{R}=0$ (red), and $\Delta \mu_{B}=0$ (blue). Parameters are p=0.39, $r=r_{1}=r_{2}=0.2$, γ=0.5.

**Figure 2:**
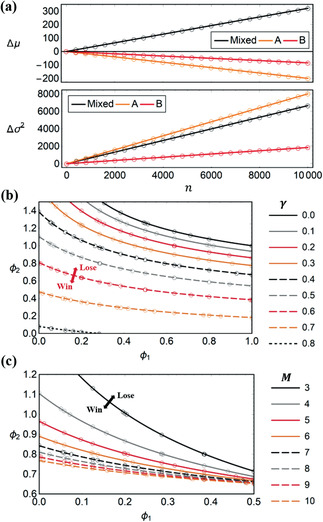
a) Plot of μ(n) and $\sigma^{2}\left(n\right)$ comparing theory (lines) and simulation (circles), realizing the strong Parrondo effect. Parameters are p=0.39, $p_{1}=0.02$, $p_{2}=0.6$, $r=r_{1}=r_{2}=0.2$, and γ=0.5. b,c) Δμ=0 fair isoclines in $\phi_{1}$‐$\phi_{2}$ parameter space for differing M and γ, comparing theory (lines) and simulation (circles). Parameters are $p_{1}=0.35$ and $r=r_{1}=r_{2}=0.1$, with M=4 for (b) and γ=0.5 for (c).

Monte Carlo simulations of the game structure were run to validate these analytical results. A comparison of Δμ and $\Delta \sigma^{2}$ from Equations (5) and (6) against simulation results for differing M is presented in **Figure** 1. The strong Parrondo effect arises when $\Delta \mu_{R}>0$ but $\Delta \mu_{A}<0$ and $\Delta \mu_{B}<0$, and the weak effect arises so long as $\Delta \mu_{R}>\Delta \mu_{A}$ and $\Delta \mu_{R}>\Delta \mu_{B}$. Phase plots of paradoxical regions and their boundaries are shown in Figure 1, and an example of μ(n) and $\sigma^{2}\left(n\right)$ exhibiting the strong paradox is shown in **Figure** **2**a. Lastly, fair boundaries (Δμ=0) as predicted by Equation (1) are shown in Figure 2b,c, alongside simulation results. Excellent agreement across all theoretical and simulation results is observed. We provide implementation details of the simulation package in Appendix C.

## 3 Generalized *M*‐Branch Game Pair

A further generalization is considered, in which game B comprises M≥3 branches $\left\{B_{1},\ldots ,B_{M}\right\}$, with branch $B_{i}$ played when c≡(i−1)modM. Each $B_{i}$ is associated with winning, drawing, and losing probabilities $p_{i}$, $r_{i}$, and $q_{i}=1-p_{i}-r_{i}$, respectively, and lose‐win ratio $\phi_{i}=q_{i}/p_{i}$. Game A remains unchanged. As before, $\eta_{p}=1$, $\eta_{q}=-1$, and $\eta_{r}=0$.

### 3.1 Conditions

Again, winning, fair, and losing conditions may first be derived. M capital states $\left\{S_{1},S_{2},\ldots ,S_{M}\right\}$ satisfying cmodM=i−1 for state $S_{i}$ are considered. By analyzing the probability of winning through M consecutive states, a simple condition of identical form can be deduced

(8) $$\begin{array}{cccc} & \text{winning} & & \text{if}\;\varphi <1\\ & \text{fair} & & \text{if}\;\varphi =1\\ & \mathrm{losing} & & \text{if}\;\varphi >1 \end{array}$$

where $\varphi =\varphi_{R}=\prod_{i=1}^{M}\phi_{i}^{\prime }$ for stochastically mixed games and $\phi_{i}^{\prime }=q_{i}^{\prime }/p_{i}^{\prime }$ are the mixed lose‐win ratios. For pure game A, γ=1, so a reduced $\varphi_{A}=\phi$ is obtained, consistent with Equation (1) for the two‐branch structure; for pure game B, γ=0, and $\varphi_{B}=\prod_{i=1}^{M}\phi_{i}$ is obtained. It is noted, furthermore, that under the conditions $s_{i}=s_{2}$ for s∈{p,r,q} and i∈[2,M], the two‐branch results $\varphi_{R}=\phi_{1}^{\prime }\phi_{2}^{\prime M-1}$ and $\varphi_{B}=\phi_{1}\phi_{2}^{M-1}$ are indeed recovered. The derivation of this result is detailed in Appendix B1.

### 3.2 Capital Statistics

Now we seek solutions for μ(n) and $\sigma^{2}\left(n\right)$. The transition matrix H is generalized to

(9) $$H_{\mathrm{ij}}=\left\{\begin{array}{cc} p_{i}^{\prime } & \text{if}\;j\equiv (i\;\mathrm{mod}\;M)+1\\ q_{i}^{\prime } & \text{if}\;i\equiv (j\;\mathrm{mod}\;M)+1\\ r_{i}^{\prime } & \text{if}\;i=j\\ 0 & \text{otherwise} \end{array}\right.$$

The payoff matrix W can again be obtained from H with $s_{i}\to \eta_{s}$ for s∈{p,r,q} and i∈{1,2}, and likewise Q=H∘W. The eigenvector equation ω=ωH characterizing the stationary distribution between states $\omega =[\omega_{1}\omega_{2}\ldots \omega_{M}]$ can likewise be converted to a coupled recurrence system, but the recurrence now involves non‐constant coefficients, so the usual method of characteristic polynomials cannot be used. Instead, a tracking method can be used on the recursion tree to yield the solution

(10) $$\begin{array}{cc} \omega_{m} & =\frac{F_{m}^{\left[1\right]}+\Lambda G_{m}^{\left[1\right]}+\delta_{1m}}{1+\sum_{i=2}^{M}F_{i}^{\left[1\right]}+\Lambda \sum_{i=2}^{M}G_{i}^{\left[1\right]}}\\ \Lambda & =\frac{p_{1}^{\prime }+q_{1}^{\prime }-p_{M}^{\prime }F_{M}^{\left[1\right]}}{p_{M}^{\prime }G_{M}^{\left[1\right]}+q_{2}^{\prime }} \end{array}$$

for m∈[1,M], and where counting functions
(11a)
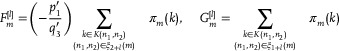

(11b) $$\begin{array}{cc} \pi_{m}\left(k\right)= & \prod_{i=1}^{\left|k\right|}\left[\delta_{1k_{i}}\left(\frac{p_{m-\sigma_{i}\left(k\right)}+q_{m-\sigma_{i}\left(k\right)}}{q_{m-\sigma_{i}\left(k\right)+1}}\right)-\delta_{2k_{i}}\left(\frac{p_{m-\sigma_{i}\left(k\right)}}{q_{m-\sigma_{i}\left(k\right)+2}}\right)\right], \end{array}$$

and $\xi_{d}\left(m\right)$ is the non‐negative solution set to the Diophantine equation $m-(n_{1}+2n_{2})=d$, parametrizable as {(m−2t−d,t):t∈Z,0≤t≤⌊(m−d)/2⌋}, $K(n_{1},n_{2})$ comprises all order permutations of $n_{1}$ ones and $n_{2}$ twos, and accumulator $\sigma_{i}\left(k\right)=\sum_{j=1}^{i}k_{j}$. The derivation of this solution is detailed in Appendix B2.

The average outcome probabilities are

(12) $$\bar{s}=\sum_{m=1}^{M}\omega_{m}s_{m}$$

for s∈{p,r,q}. Substituting $\bar{p}$ and $\bar{q}$ into Equation (5) yields Δμ and μ(n) for this generalized M‐branch structure. Obtaining $\sigma^{2}\left(n\right)$ likewise necessitates computing the fundamental matrix Z. A similar procedure as that for the two‐branch structure can be used, but again, as the recurrence is non‐constant, a tracking method has to be employed. The derivation and full solution for Z is given in Appendix B3. Expanding and simplifying d=ωQ(Z−Ω)Qℓ gives

(13) $$\begin{array}{cc} d= & \;\sum_{i=1}^{M}\sum_{j=1}^{M}\left(p_{i_{-}}^{\prime }\omega_{i_{-}}-q_{i_{+}}^{\prime }\omega_{i_{+}}\right)\left(Z_{ij}-\omega_{j}\right)\left(p_{j}^{\prime }-q_{j}^{\prime }\right) \end{array}$$

Substituting d into Equation (6) completes the closed‐form solution for $\sigma^{2}\left(n\right)$. In summary, we have closed‐form solutions for parameter space boundaries in Equation (8), and capital statistics μ(n) and $\sigma^{2}\left(n\right)$ in Equations (5), (6), (12), and (13). Again, since μ(n) and $\sigma^{2}\left(n\right)$ are constructed from exact steady‐state Δμ and $\Delta \sigma^{2}$ and neglect transients, an accuracy of O(1/n) is expected.

### 3.3 Validation

**Figure 3:**
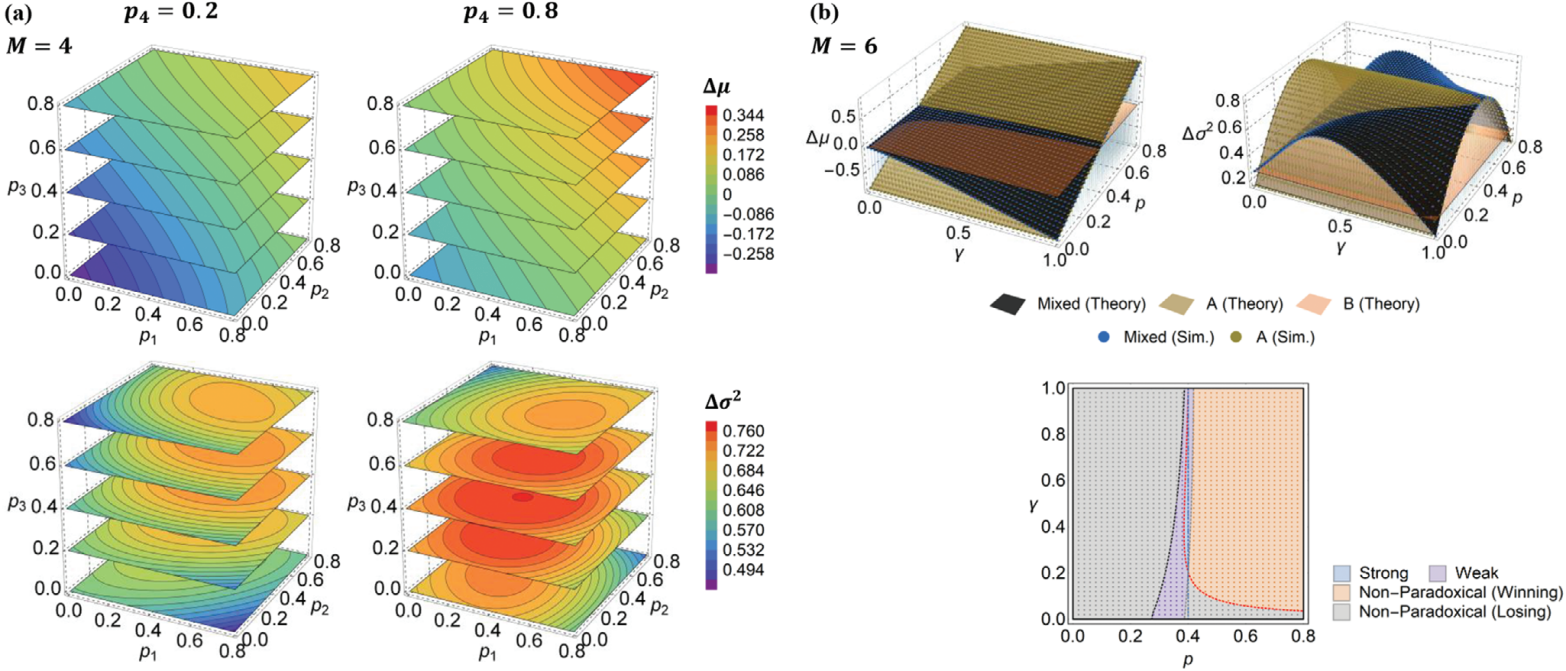
a) Theoretical results on Δμ and $\Delta \sigma^{2}$ across $p_{i}$ parameter space for M=4. The full parameter space is 4D and cannot be directly illustrated; slices across $p_{i}-p_{2}-p_{3}$ space are shown, for two values of $p_{4}$. To preserve visual clarity, simulation results are not plotted, but relative error is <0.1% throughout. Parameters are p=0.39, $r=r_{i}=0.2$ for i∈[1,4], and γ=0.5. b) Δμ and $\Delta \sigma^{2}$ from theory and simulations across p–γ parameter space, and phase plot, where background colors represent theory and dots represent simulation. Dotted lines denote theoretical boundaries, for $\Delta \mu_{R}=\Delta \mu_{B}$ (black), $\Delta \mu_{R}=\Delta \mu_{A}$ (gray), $\Delta \mu_{R}=0$ (red), and $\Delta \mu_{A}=0$ (blue). Parameters are $r=r_{i}=0.2$, $p_{1}=0.02$, and $p_{i}=0.5$ for i∈[2,6].

Monte Carlo simulations were likewise run to validate these theoretical results. Contour plots of Δμ and $\Delta \sigma^{2}$ as computed from Equations (12) and (13) across *p_*i*_* parameter space for M=4 are illustrated in **Figure** 3a, for which simulation results match to excellent accuracy. A comparison of Δμ and $\Delta \sigma^{2}$ from theory and simulations across p–γ parameter space is also given in Figure 3b. The theoretical capital statistics and phase boundaries match simulations extremely well. Implementation details of the simulations are provided in Appendix C.

## 4 General Outlook

This paper has presented much‐sought explicit closed‐form solutions for capital statistics and parameter space boundaries for a family of generalized Parrondo's games, of which the canonical game pair^[^ ^7^ ^]^ is a special case. The results are exact in steady‐state conditions, and of O(1/n) accuracy when initial transients are present; the effect of transients is dependent on initial conditions and game configuration, and will manifest as a small offset in capital statistics against steady‐state predictions. Importantly, the solutions accommodate arbitrary modulus M and probability configurations; this work hence reports a fully general closed‐form solution for single‐agent capital‐dependent games. In comparison to usual Markov‐chain propagation calculations in predicting capital statistics, these solutions enable computational cost improvements in the treatment of related models (see Appendix D).

Recent developments in physics of living systems^[^ ^20^, ^21^, ^36^ ^]^ and noise‐induced phenomena^[^ ^49^, ^50^, ^51^, ^52^, ^53^, ^54^, ^55^, ^56^ ^]^ have renewed interest in stochastic ratchet‐type mechanisms and the broader Parrondo framework—the methodology developed here is general to the multitude of Parrondo‐type model variants, so long as modelable as discrete‐time Markov chains (of arbitrary order), and can enable a paradigm shift from the current reliance on numerical simulations toward analytical methods. In particular, the theoretical treatment of ratcheting eco‐evolutionary and biomechanical models may benefit from the presented results. It may be of interest, in the future, to extend a similar analysis to history‐dependent game structures or multi‐agent games on arbitrary graph topologies.

## Conflict of Interest

The authors declare no conflict of interest.

## Supporting information

Supporting Information
