# Supplementary material for: Generalized Solutions of Parrondo's Games
Source: Adv Sci (Weinh). 2020 Nov 7;7(24):2001126. doi: 10.1002/advs.202001126 (PMC7740106; doi:10.1002/advs.202001126)
Supplement: Supplementary file 1 — Supporting Information [file ADVS-7-2001126-s001.pdf]

© 2020 Wiley-VCH GmbH

ADVANCED SCIENCE
Open Access

Supporting Information

for *Adv. Sci.*, DOI: 10.1002/advs.202001126

Generalized Solutions of Parrondo's Games

*Jin Ming Koh and Kang Hao Cheong**

Supporting Information: Generalized Solutions of Parrondo's Games

Jin Ming Koh[1,2] and Kang Hao Cheong[1,3,*]

[1]*Singapore University of Technology and Design (SUTD), 8 Somapah Rd, S487372, Singapore*
[2]*California Institute of Technology, Pasadena, CA 91125, USA*
[3]*SUTD-Massachusetts Institute of Technology International Design Centre, Singapore*

Appendix A: Three-State Game Pair

A1. Conditions

Let f_z be the probability that the capital ever reaches zero given a starting amount of $z \in \mathbb{Z}$ units. It is then consequent of Markov chain theory [1, 2] that either $f_z = 1 \,\forall\, z \geq 0$, reflecting that the game is fair or losing, or that $f_z < 1 \,\forall\, z > 0$, in which case it is possible that the capital will grow indefinitely and the game is winning. The set $\{f_z\}$ is the minimal non-negative solution to the recurrence equation

$$f_{Mk} = p_1' f_{Mk+1} + r_1' f_{Mk} + q_1' f_{Mk-1}, \tag{A1a}$$

$$f_{Mk+l} = p_2' f_{Mk+l+1} + r_2' f_{Mk+l} + q_2' f_{Mk+l-1}, \tag{A1b}$$

where $k \geq 1$, $l \in \{1, ..., M-1\}$, and boundary condition $f_0 = 1$ applies. Solving Eq. (A1b) yields a general solution $f_{Mk+l} = a_1 \phi_2'^{l} + a_2$, where

$$a_1 = \frac{f_{Mk} - f_{M(k+1)}}{1 - \phi_2'^M}, \tag{A2a}$$

$$a_2 = \frac{f_{M(k+1)} - f_{Mk}\phi_2'^M}{1 - \phi_2'^M}. \tag{A2b}$$

Substituting this solution into Eq. (A1a) and solving yields $f_{Mk} = a_3(\varphi^k - 1) + 1$ with $\varphi \equiv \phi_1' \phi_2'^{M-1}$. If $\varphi \geq 1$, the minimal non-negative solution occurs when $a_3 = 0$, so $f_{Mk} = 1 \,\forall\, k \geq 0$; otherwise, the minimal non-negative solution occurs when $a_3 = 1$, thus $f_{Mk} = \varphi^k < 1 \,\forall\, k > 0$. It is hence summarized that $f_{Mk} = \min\{1, \varphi^k\}$ for stochastically mixed games. This leads to the result in Eq. (1) of the main paper.

A2. Stationary Distribution

The eigenvalue equation $\omega = \omega H$ produces the set of equations

$$\omega_1 = \omega_1 r_1' + \omega_2 q_2' + \omega_M p_2', \tag{A3a}$$

$$\omega_2 = \omega_1 p_1' + \omega_2 r_2' + \omega_3 q_2', \tag{A3b}$$

$$\omega_m = \omega_{m-1} p_2' + \omega_m r_2' + \omega_{m+1} q_2', \tag{A3c}$$

$$\omega_M = \omega_1 q_1' + \omega_{M-1} p_2' + \omega_M q_2', \tag{A3d}$$

where $m \in [3, M-1]$. Eq. (A3c) is first solved with ω_2 and ω_3 as boundary conditions. Invoking Eqs. (A3a) and (A3b) eliminates ω_2 and ω_3, and the normalization constraint $\sum_{m=1}^{M} \omega_m = 1$ sets ω_1. This yields the solution given in Eq. (3) of the main paper.

A3. Capital Distribution

Suppose that out of n rounds, n_+ result in wins, n_0 result in draws, and n_- result in losses. At steady-state, the average outcome probabilities are $\bar{s} = \omega_1 s_1' + (1-\omega_1) s_2'$ for $s \in \{p, q, r\}$, where ω_1 is the stationary distribution of capital state S_1. The distribution $\mathcal{P}_n(k)$ representing the probability of having $k \in \mathbb{Z} \cap [-n, n]$ capital on round n can thus be written

$$\mathcal{P}_n(k) = \sum \frac{n!}{n_+! n_0! n_-!} \bar{p}^{n_+} \bar{r}^{n_0} \bar{q}^{n_-}, \tag{A4}$$

where the summation occurs over the solution set of simultaneous Diophantine equations $n_+ + n_0 + n_- = n$ and $n_+ - n_- = k$. Such a solution set can be parametrized as $(n_+, n_0, n_-) = (u + (|k|+k)/2, n - |k| - 2u, u + (|k|-k)/2$ where $u \in \mathbb{Z}$ and $0 \leq u \leq \lfloor (n-k)/2 \rfloor)$. The summation in Eq. (A4) is thus over u, enabling the closed-form calculation of $\mathcal{P}_n(k)$.

The expected capital $\mu(n)$ can be computed from this explicit capital distribution as

$$\mu(n) = \sum_{k=-n}^{n} k P_n(k) = (\bar{p} - \bar{q})\, n, \tag{A5}$$

which is identical to the result obtained in Eq. (5) of the main paper.

A4. Fundamental Matrix

We have $Z = (I - H + \Omega)^{-1}$, but to simplify calculations, the identity $Z(I - H) = I - \Omega$ is used. This produces the set of equations

$$\delta_{i1} - \omega_1 = Z_{i1}(1 - r_1') - Z_{i2} q_2' - Z_{iM} p_2', \tag{A6a}$$

$$\delta_{i2} - \omega_2 = -Z_{i1} p_1' + Z_{i2}(1 - r_2') - Z_{i3} q_2', \tag{A6b}$$

$$\delta_{ij} - \omega_j = -Z_{i(j-1)} p_1' + Z_{ij}(1 - r_2') - Z_{i(j+1)} q_2', \tag{A6c}$$

$$\delta_{iM} - \omega_M = -Z_{i1} q_1' - Z_{i(M-1)} p_2' + Z_{iM}(1 - r_2'), \tag{A6d}$$

where $i \in [1, M]$ and $j \in [3, M-1]$. Eq. (A6c) is first solved with Z_{i2} and Z_{i3} as boundary conditions. Invoking Eqs. (A6a) and (A6b) eliminates Z_{i2} and Z_{i3}, and the normalization constraint $\sum_{j=1}^{M} Z_{ij} = 1$ sets Z_{i1}. This yields

* Corresponding author. E-mail: kanghao_cheong@sutd.edu.sg

$$\begin{aligned}Z_{i1} =\ & (p_2'/\rho)\left\{2q_2'^M\left(p_2'-q_2'\right)^2\left\{q_2'\left[1-i-\left(p_2'-q_2'\right)\right]+\phi_2'^{-M}\left[Mp_2'\phi_2'^i-q_2'\left[M+1-i-\left(p_2'-q_2'\right)\right]\right]\right\}\right.\\&-2\alpha p_2'\phi_2'^{-M}\left[M^2q_2'^M\left(p_2'-q_2'\right)^2-p_2'q_2'\left(p_2'^M-q_2'^M\right)\left(1-\phi_2'^M\right)\right]\\&\left.+M\beta q_2'\left(p_2'-q_2'\right)\left\{q_2'^M\left[M\left(p_2'-q_2'\right)+\left(p_2'+q_2'\right)\right]+p_2'^M\left[\left(M-1\right)p_2'-\left(M+1\right)q_2'\right]\right\}\right\},\end{aligned} \tag{A7a}$$

$$\begin{aligned}Z_{i2} =\ & \left\{p_2'^M\left[p_2'\phi_2^i\left(p_2'-q_2'\right)-q_2'\phi_2'^M\left(p_2'-q_2'\right)\right]+Z_{i1}\left(1-\phi_2'\right)\left\{p_1'p_2'^Mq_2'^2+p_2'q_2'^M\left[p_2'q_1'-q_2'\left(p_1'+q_1'\right)\right]\right\}\right.\\&\left.+\alpha p_2'\left[Mp_2'^Mq_2'-p_2'q_2'^M-\left(M-1\right)p_2'^{M+1}\right]+\beta q_2'\left\{q_2'^M\left[M\left(p_2'-q_2'\right)+q_2'\right]-p_2'^Mq_2'\right\}\right\}/\\&q_2'^2\left(p_2'-q_2'\right)\left(p_2'^M-q_2'^M\right),\end{aligned} \tag{A7b}$$

$$\begin{aligned}\rho =\ & 2\left(p_2'-q_2'\right)^2\left\{p_2'^Mq_2'\left\{p_2'\left[\left(M-1\right)p_1'+p_2'+q_1'\right]-q_2'\left(Mp_1'+p_2'\right)\right\}\right.\\&\left.-p_2'q_2'^M\left\{Mq_1'\left(p_2'-q_2'\right)+q_2'\left[\left(p_2'-q_2'\right)-\left(p_1'-q_1'\right)\right]\right\}\right\}.\end{aligned} \tag{A7c}$$

We have $Z_{ij}=Z_{i1}$ for $j=1$, and for $2\le j\le M$, the general solution is

$$\begin{aligned}Z_{ij} =&\Big\{Z_{i1}p_1'\left(p_2'-q_2'\right)\left(1-\phi_2'^{2-j}\right)-Z_{i2}q_2'\left(p_2'-q_2'\right)\left(1-\phi_2'^{1-j}\right)+\alpha\left\{p_2'\phi_2'^{-1}+\phi_2'^{-j}\left[p_2'\left(j-2\right)-q_2'\left(j-1\right)\right]\right\}\\&-\beta\left[\left(j-1\right)p_2'-\left(j-2\right)q_2'-p_2'\phi_2'^{2-j}\right]+\left(p_2'-q_2'\right)\left(1-\phi_2'^{-R(j-i)}\right)\Big\}\Big/\left(p_2'-q_2'\right)^2,\end{aligned} \tag{A8}$$

where $R(x)$ is the unit ramp function.

Appendix B: Three-state M-branch game pair

B1. Conditions

Every M consecutive states is termed a tier. Winning a tier necessitates winning across all M branches; furthermore, an arbitrary number of losses l_i at each state S_i is allowed, so long as there is a corresponding number of wins to compensate. The probability of winning and losing a tier, respectively $\widetilde{p}$ and $\widetilde{q}$, is thus

$$\widetilde{s}=\prod_{i=1}^{M}s_i'\cdot\sum_{l_1,\ldots,l_M=0}^{\infty}\left[\prod_{i=1}^{M}\left(q_i'p_{i-1}'\right)^{l_i}\right], \tag{B1}$$

where $s_i'=\gamma s+(1-\gamma)s_i$ for $s\in\{p,r,q\}$ and $i\in[1,M]$ are the mixed transition probabilities. The game is winning, fair, and losing when $\widetilde{p}>\widetilde{q}$, $\widetilde{p}=\widetilde{q}$, and $\widetilde{p}<\widetilde{q}$ respectively. Cancellation of terms yield the simplistic condition in Eq. (8) of the main paper.

B2. Stationary Distribution

The eigenvector equation $\omega=\omega H$ produces the set of equations

$$\omega_1=\omega_1r_1'+\omega_2q_2'+\omega_Mp_M', \tag{B2a}$$
$$\omega_m=\omega_{m-1}p_{m-1}'+\omega_mr_m'+\omega_{m+1}q_{m+1}', \tag{B2b}$$
$$\omega_M=\omega_1q_1'+\omega_{M-1}p_{M-1}'+\omega_Mr_M', \tag{B2c}$$

where $m\in[2,M-1]$. But, as the recurrence in Eq. (B2b) involves non-constant coefficients, the usual method of solving the characteristic polynomial cannot be used. Instead, a tracking method can be used on the recursion tree to arrive at

$$\omega_m=F_m^{[1]}\omega_1+G_m^{[1]}\omega_2, \tag{B3}$$

where F and G are counting functions as written in the main paper. Invoking Eq. (B2a) to eliminate ω_2 and the normalization constraint $\sum_{m=1}^{M}\omega_m=1$ to set ω_1 then yields the solution for ω_m as presented in Eq. (10) of the main paper.

B3. Fundamental Matrix

Again, the identity $Z(I-H)=I-\Omega$ is used. This produces the set of equations

$$\delta_{i1}-\omega_1=Z_{i1}(1-r_1')-Z_{i2}q_2'-Z_{iM}p_M', \tag{B4a}$$
$$\delta_{ij}-\omega_j=-Z_{i(j-1)}p_{j-1}'+Z_{ij}(1-r_j')-Z_{i(j+1)}q_{j+1}', \tag{B4b}$$
$$\delta_{iM}-\omega_M=-Z_{i1}q_1'-Z_{i(M-1)}p_{M-1}'+Z_{iM}(1-r_M'), \tag{B4c}$$

where $i\in[1,M]$ and $j\in[2,M-1]$. As the recurrence in Eq. (B4b) is non-constant, the method of characteristic polynomials cannot be applied. The recurrence tree is tracked to give

$$Z_{ij}=F_j^{[1]}Z_{i1}+G_j^{[1]}Z_{i2}+T_j^{[3]}-G_j^{[i]}/q_{i+1}', \tag{B5}$$

where

$$T_m^{[l]}=\sum_{\substack{k\in K(n_1,n_2)\\(n_1,n_2)\in\zeta_l(m)}}\left(\frac{\omega_{m-\sigma_{|k|}(k)-1}}{q_{m-\sigma_{|k|}(k)}'}\right)\pi_m(k), \tag{B6a}$$

$$\zeta_d(m)=\bigcup_{i=d}^{m}\xi_i(m). \tag{B6b}$$

Invoking Eq. (B4a) to eliminate Z_{i2} and the normalization constraint $\sum_{j=1}^{M}Z_{ij}=1$ to set Z_{i1} then yields the solution

$$\begin{aligned}Z_{ij}&=\left(F_j^{[1]}+\Lambda G_j^{[1]}+\delta_{1j}\right)Z_i^*-\varrho_iG_j^{[1]}+T_j^{[3]}-\frac{G_j^{[i]}}{q_{i+1}'},\\Z_i^*&=\frac{1+\frac{1}{q_{i+1}'}\sum_{j=i}^{M}G_j^{[i]}-\sum_{j=2}^{M}T_j^{[3]}+\varrho_i\sum_{j=2}^{M}G_j^{[1]}}{1+\sum_{j=2}^{M}F_j^{[1]}+\Lambda\sum_{j=2}^{M}G_j^{[1]}},\\\varrho_i&=\frac{p_M\left(q_{i+1}'T_M^{[3]}-G_M^{[i]}\right)-q_{i+1}'\omega_1}{\left(p_M'G_M^{[1]}+q_2'\right)q_{i+1}'}.\end{aligned} \tag{B7}$$

Appendix C: Numerical Simulations

Double-precision numerical Monte Carlo simulations of the presented game structures were written in *Java 11*. The stochastic mixing of games and selection of outcomes at each game round were performed using native random-number generators, provided by the `SplittableRandom` class to facilitate parallelization. All statistical results were averaged over at least 10^6 trials where applicable to suppress noise; simulations were run on a 16-core machine. Visualization of the simulation data and comparison against theory were performed in *Mathematica 12*.

Appendix D: Computational Complexity

A technical motivation for pursuing the analytical capital statistics solutions in the main paper is to bypass the computational cost of Markov-chain calculations in making predictions. We analyze this as follows. The basic approach to compute capital statistics is to propagate the initial capital distribution vector using the full transition matrix. For n game rounds, since the capital may be changed by a unit amount each round, the capital distribution vector will be $\mathcal{O}(n)$ long, and the transition matrix will be $\mathcal{O}(n \times n)$ large. Therefore arriving at the final capital distribution will be $\mathcal{O}(n^3)$ expensive, followed by post-processing to compute statistics of interest. This is unwieldy, as n can be large, say, $\sim \mathcal{O}(10^6)$ in some practical applications.

As was carried out in this paper, and is common in literature, one may contract the matrix to $M \times M$ by considering capital states modulo M, and accordingly the time complexity to compute the final distribution vector may be improved to $\mathcal{O}(nM^2)$. It is possible to reduce this further, but at least some matrix operations are required, such as spectral decomposition, inverses, and multiplication, so $\mathcal{O}(M^3)$ is a lower bound. In this work M is general, and can be arbitrarily large, so this is not entirely satisfactory. Having explicit analytical, or otherwise closed-form, solutions for capital statistics allows the side-stepping of these calculations to give nearly $\mathcal{O}(1)$ theoretical evaluations, a huge advancement.

[1] G. P. Harmer, D. Abbott, P. G. Taylor, and J. M. R. Parrondo, AIP Conf. Proc **511**, 189 (2000).

[2] J. G. Kemeny and J. L. Snell, *Markov Chains* (Springer-Verlag, New York, 1976).
